# Extracellular ATP inhibits twitching motility-mediated biofilm expansion by *Pseudomonas aeruginosa*

**DOI:** 10.1186/s12866-015-0392-x

**Published:** 2015-03-01

**Authors:** Laura M Nolan, Rosalia Cavaliere, Lynne Turnbull, Cynthia B Whitchurch

**Affiliations:** The ithree institute, University of Technology Sydney, PO Box 123, Broadway, NSW 2007 Australia

**Keywords:** 3’,5’-adenosine triphosphate, ATP, Twitching motility, Type IV pili, Tfp, t4p, eATP

## Abstract

**Background:**

*Pseudomonas aeruginosa* is an opportunistic pathogen that exploits damaged epithelia to cause infection. Type IV pili (tfp) are polarly located filamentous structures which are the major adhesins for attachment of *P. aeruginosa* to epithelial cells. The extension and retraction of tfp powers a mode of surface translocation termed twitching motility that is involved in biofilm development and also mediates the active expansion of biofilms across surfaces. Extracellular adenosine triphosphate (eATP) is a key “danger” signalling molecule that is released by damaged epithelial cells to alert the immune system to the potential presence of pathogens. As *P. aeruginosa* has a propensity for infecting damaged epithelial tissues we have explored the influence of eATP on tfp biogenesis and twitching motility-mediated biofilm expansion by *P. aeruginosa*.

**Results:**

In this study we have found that eATP inhibits *P. aeruginosa* twitching motility-mediated expansion of interstitial biofilms at levels that are not inhibitory to growth. We have determined that eATP does not inhibit expression of the tfp major subunit, PilA, but reduces the levels of surface assembled tfp. We have also determined that the active twitching zone of expanding *P. aeruginosa* interstitial biofilms contain large quantities of eATP which may serve as a signalling molecule to co-ordinate cell movements in the expanding biofilm. The inhibition of twitching motility-mediated interstitial biofilm expansion requires eATP hydrolysis and does not appear to be mediated by the Chp chemosensory system.

**Conclusions:**

Endogenous eATP produced by *P. aeruginosa* serves as a signalling molecule to co-ordinate complex multicellular behaviours of this pathogen. Given the propensity for *P. aeruginosa* to infect damaged epithelial tissues, our observations suggest that eATP released by damaged cells may provide a cue to reduce twitching motility of *P. aeruginosa* in order to establish infection at the site of damage. Furthermore, eATP produced by *P. aeruginosa* biofilms and by damaged epithelial cells may play a role in *P. aeruginosa* pathogenesis by inducing inflammatory damage and fibrosis. Our findings have significant implications in the development and pathogenesis of *P. aeruginosa* biofilm infections.

**Electronic supplementary material:**

The online version of this article (doi:10.1186/s12866-015-0392-x) contains supplementary material, which is available to authorized users.

## Background

*Pseudomonas aeruginosa* is a Gram negative bacterium found throughout the environment and is an opportunistic pathogen of a wide variety of eukaryotic hosts [1,2]. *P. aeruginosa* causes acute and chronic infections in immunocompromised humans and is the major contributor to the morbidity and mortality of individuals with cystic fibrosis [3,4]. *P. aeruginosa* establishes infections at sites of epithelial tissue damage such as those encountered in patients with severe burns, mechanical ventilation, or corneal damage due to contact lens use [5,6]. *P. aeruginosa* can establish chronic infections which are associated with the formation of complex, surface-associated communities termed biofilms [7].

Type IV pili (tfp) are the major adhesins of *P. aeruginosa* that promote attachment to host epithelial cells. These polar, filamentous surface structures also mediate a form of surface translocation termed twitching motility that occurs through the extension, surface binding and retraction of tfp [8]. The active expansion of *P. aeruginosa* biofilms is a complex, multicellular, collective behaviour that is mediated by twitching motility [9,10]. A number of host derived signals including mucin, serum albumin, oligopeptides, phosphatidylcholine (PC), lactoferrin and low levels of available iron have been shown to stimulate twitching motility-mediated biofilm expansion by *P. aeruginosa* [11-15]. Interestingly, mucin, serum albumin, lactoferrin, and low iron have also been shown to inhibit the formation of sessile biofilms that form under fully hydrated conditions [12-15]. It has been suggested that the effects of these host-derived compounds may provide a protective advantage to the host by inhibiting the ability of *P. aeruginosa* to form resistant, sessile biofilms, and thus giving the immune system a better chance of clearing the infection [14].

Another host-derived signalling molecule, extracellular 3’,5’-adenosine triphosphate (eATP) is rapidly released in high levels (5–10 mM) by stressed or injured epithelial cells to alert the host to the presence of invading pathogens, resulting in recruitment of immune system factors to clear the infection [16,17]. As *P. aeruginosa* displays a propensity for infecting damaged epithelial tissues [5] it is therefore likely to encounter high levels of eATP at the site of damaged epithelium where it initiates infection. In this study we have explored the possibility that eATP may be a host-derived molecule that affects twitching motility in *P. aeruginosa* and have found that eATP inhibits *P. aeruginosa* twitching motility-mediated expansion of interstitial biofilms at levels that are not inhibitory to growth.

## Results

### Extracellular ATP inhibits twitching motility

**Figure 1:**
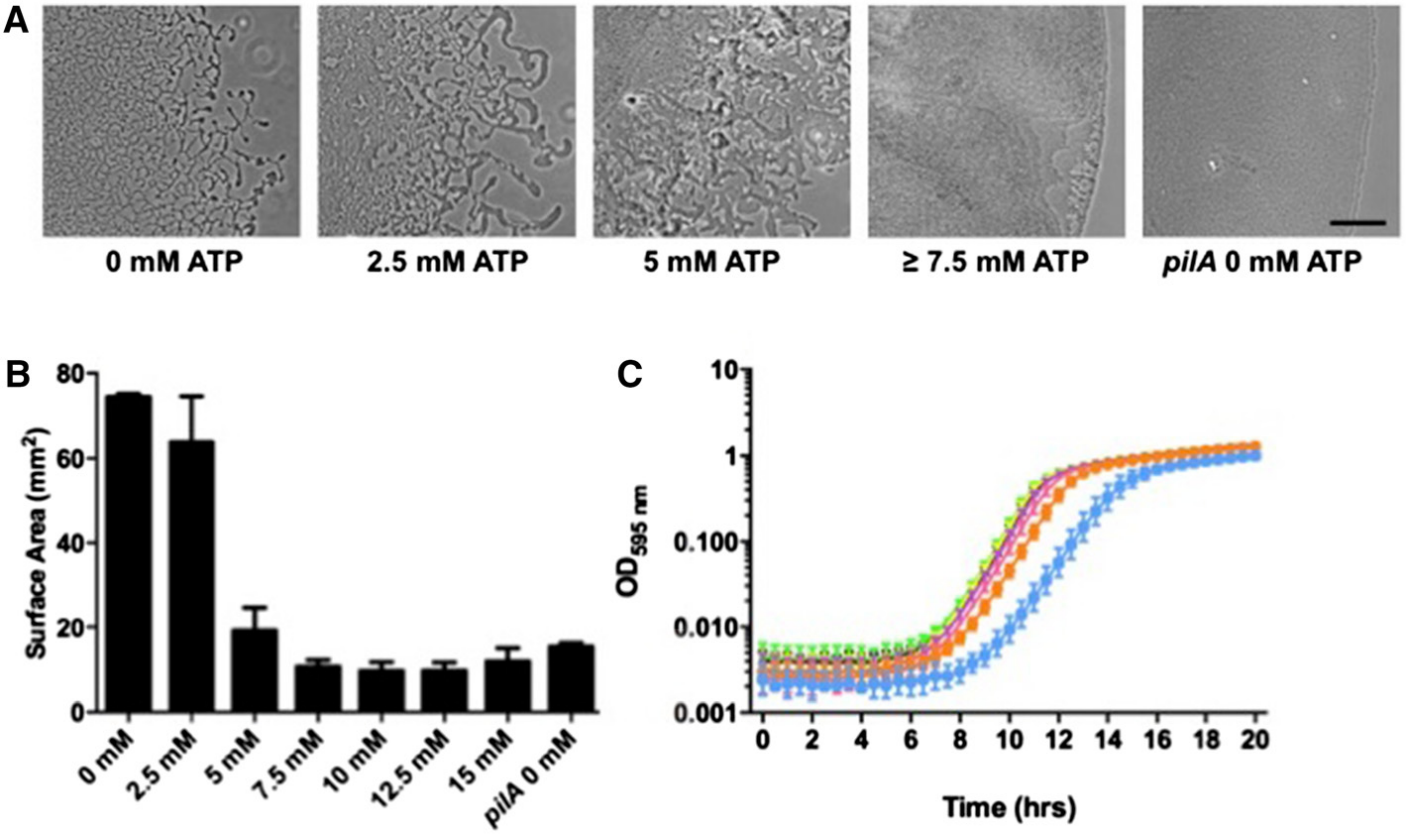
**Extracellular ATP inhibits twitching motility-mediated expansion of** ***P. aeruginosa*** **interstitial biofilms. (A)** Representative light microscopy images of *P. aeruginosa* PAK and PAK*pilA* interstitial biofilms formed at the interface between a microscope slide coated in solidified nutrient media supplemented with a range of eATP concentrations and a coverslip after incubation at 37°C for 16 h. Scale 50 μm. **(B)** Surface areas of the interstitial biofilms formed by *P. aeruginosa* PAK and PAK*pilA* at the interface between a microscope slide coated in solidified nutrient media supplemented with a range of eATP concentrations and a coverslip after incubation at 37°C for 16 h. Mean ± SD from three independent experiments run in duplicate are presented. **(C)** Planktonic growth of *P. aeruginosa* PAK at 37°C over 20 h in media supplemented with 0 mM ATP (black), 2.5 mM ATP (green), 5 mM ATP (yellow), 7.5 mM ATP (magenta), 10 mM ATP (pink), 12.5 mM ATP (orange) or 15 mM ATP (blue). Mean ± SD from three independent experiments each consisting of five technical replicates is presented.

To determine if eATP influences twitching motility-mediated expansion of interstitial biofilms by *P. aeruginosa*, we utilised our interstitial twitching motility assay [18] to examine the effect of incorporating eATP into the solidified nutrient media. In this assay *P. aeruginosa* cells are inoculated at the interface between a glass coverslip and a microscope slide coated in nutrient media that has been solidified with gellan gum. Under these conditions, twitching motility mediates rapid, active biofilm expansion at the interface between the glass coverslip and solidified nutrient media [9,10]. The resultant biofilm is imaged using phase-contrast microscopy and quantitated using image analysis tools. To determine if eATP affects *P. aeruginosa* PAK twitching motility-mediated expansion of interstitial biofilms, varying concentrations of eATP (0 mM–15 mM) were incorporated into the nutrient media. After 16 h incubation at 37°C the resulting biofilms were imaged (Figure 1A) and the surface area covered by the biofilms calculated (Figure 1B). These analyses revealed that eATP concentrations of 5 mM and above dramatically reduced twitching motility-mediated biofilm expansion to levels equivalent to that of the non-twitching tfp pilin mutant PAK*pilA*::TcR (Figure 1B) [9,19]. Furthermore, microscopic examination of the interstitial biofilms (Figure 1A) shows that at eATP concentrations of 7.5 mM and above, the resultant biofilms resemble those of the non-twitching PAK*pilA*::Tc^R^ strain as they no longer form the leading-edge rafts and lattice-like trail network which are characteristic micromorphological features of wild-type *P. aeruginosa* interstitial biofilms in this assay [9,10] (Figure 1A). Interestingly in the presence of 2.5 mM and 5 mM eATP the interstitial biofilms had very large leading edge rafts and had lost the ability to self-organise into the characteristic refined trail network (Figure 1A).

We determined if the reduction in interstitial biofilm expansion might be a consequence of reduced growth in the presence of eATP. The planktonic growth of *P. aeruginosa* PAK in nutrient media supplemented with eATP at concentrations of 0–15 mM was measured by recording OD_595nm_ over a 20 h period (Figure 1C). These assays show that up to 10 mM eATP does not affect planktonic growth, while at 12.5 mM and 15 mM ATP the planktonic growth rate was slightly reduced. These observations indicate that addition of exogenous eATP at concentrations between 2.5 mM and 10 mM specifically inhibits twitching motility-mediated biofilm expansion of *P. aeruginosa* while planktonic growth remains unaffected.

To further understand the influence of addition of eATP to twitching motility-mediated expansion of interstitial biofilms, we performed high resolution time-lapse imaging of expanding biofilms in the presence of 0 mM, 2.5 mM, 5 mM and 7.5 mM eATP (Additional file 1: Movie 1). In the absence of eATP cells within the trails behind the leading-edge rafts maintain a lattice-like network, which is characteristic of twitching motility-mediated interstitial biofilm expansion [9,10]. However, at eATP concentrations of 2.5 mM and 5 mM cells within the trails are no longer able to maintain this lattice-like network. While most of the cells are still moving in the presence of 2.5 mM and 5 mM eATP, the individual cells have lost the ability to coordinate their movements and instead appear to be moving randomly which inhibits the ability of *P. aeruginosa* cells to effectively undergo twitching motility-mediated interstitial biofilm expansion. At an eATP concentration of 7.5 mM cells are non-twitching and form a biofilm edge which is similar to that of the non-twitching PAK*pilA*::Tc^R^ strain [9].

### Extracellular ATP decreases surface tfp levels

**Figure 2:**
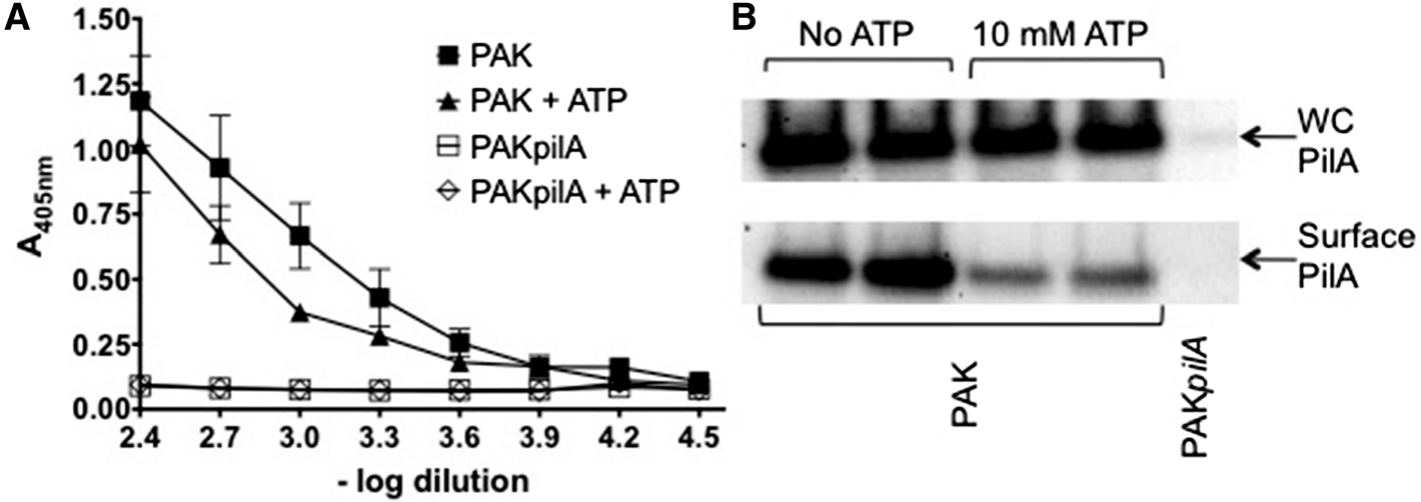
**Extracellular ATP decreases levels of surface-assembled tfp. (A)** ELISA of surface assembled tfp on whole cells from *P. aeruginosa* strains PAK and PAK*pilA* obtained from overnight (20 h) confluent lawns grown at 37°C on LB agar plates containing 0 mM or 10 mM ATP. Levels of surface assembled tfp were detected with anti-PilA anti-serum. Data are presented as the mean ± SD and are representative of results obtained from four independent experiments run in triplicate. **(B)** Immunoblot of pilin (PilA) detected in whole cell (WC) preparations (top panel) and in sheared surface tfp preparations (bottom panel) of strains PAK (lanes 1 – 4) and PAK*pilA* (lane 5) obtained from overnight (20 h) confluent lawns grown at 37°C on LB agar plates containing 0 mM or 10 mM ATP. Equal amounts of cells were used in each assay. The results shown in the immunoblots are representative of results obtained in four independent experiments run in duplicate.

Our observations have indicated that 10 mM eATP causes twitching motility to cease but does not affect planktonic growth. One mechanism via which this may occur is through modulation of tfp production. To investigate this, levels of surface assembled tfp of wild-type PAK cultured on agar containing 0 mM or 10 mM eATP was assessed by ELISA of whole cells and Western analysis of whole cells and sheared tfp using antisera against the major pilin subunit PilA (Figure 2A, B). These analyses indicate that levels of surface assembled tfp are reduced in the presence of 10 mM eATP (Figure 2A, B) whereas the levels of cell-associated pilin are not altered by 10 mM eATP (Figure 2B). These observations suggest that eATP does not affect tfp pilin expression but rather is likely to be reducing tfp assembly or increasing tfp retraction resulting in the observed reduction of surface-assembled tfp levels.

### The leading edge of actively expanding *P. aeruginosa* biofilms contains eATP

Our time-lapse microscopy of *P. aeruginosa* twitching motility-mediated interstitial biofilm expansion in the presence of exogenous eATP indicates that addition of eATP at 2.5–5 mM into the solidified nutrient media appears to inhibit co-ordinated cell movements while an eATP concentration of 7.5 mM causes cell movement to cease (Additional file 1: Movie 1). A possible explanation for this phenomenon is that a gradient of eATP within the active migration zone acts as a directional signal for cells to enable them to differentiate which direction to travel to ensure that overall motility is directed toward virgin territory and away from the mature biofilm. At higher concentrations, eATP may also be acting as a cue to indicate that a cell is no longer located at the leading edge and that movement should cease. Interestingly, Semmler et al. (1999) noted that bacterial cells in the mature regions of the biofilm appear to settle [9], which would be consistent with higher eATP concentrations in this region inducing the cessation of twitching motility.

It has been previously reported that *P. aeruginosa* secretes eATP in broth culture [20,21]. To explore the possibility that *P. aeruginosa* produces endogenous eATP in the actively migrating zone at the edge of actively expanding biofilms cultured on solidified nutrient media, we resuspended cells from the actively migrating edge of a colony biofilm and measured the concentration of ATP in the cell-free supernatant. These assays indicate that the active zone of twitching motility at the edge of expanding *P. aeruginosa* PAK colony biofilms contains about 3 mM eATP. Interestingly, this is significantly higher than eATP concentrations measured in planktonic cultures which were measured in the nm-μm range [20,21]. The higher eATP concentrations that we measured in the active twitching zone of *P. aeruginosa* colony biofilms may be a consequence of increased secretion and/or reduced diffusion. The ~3 mM concentration of eATP measured in the active twitching zone of the colony biofilm likely reflects the average of the eATP concentrations across the region measured. Our assay was not sufficiently sensitive to determine if there is a gradient of eATP within this zone and if so what was its range. However, given that we have observed that addition of 2.5–10 mM exogenous eATP into the nutrient media appears to inhibit co-ordinated directional movement during twitching motility mediated biofilm expansion and ultimately causes the cells to cease movement, this average value of 3 mM would be consistent with the levels of exogenous eATP confounding an endogenous eATP gradient within the actively migrating zone.

### eATP gradients inhibit twitching motility in approaching biofilms

**Figure 3:**
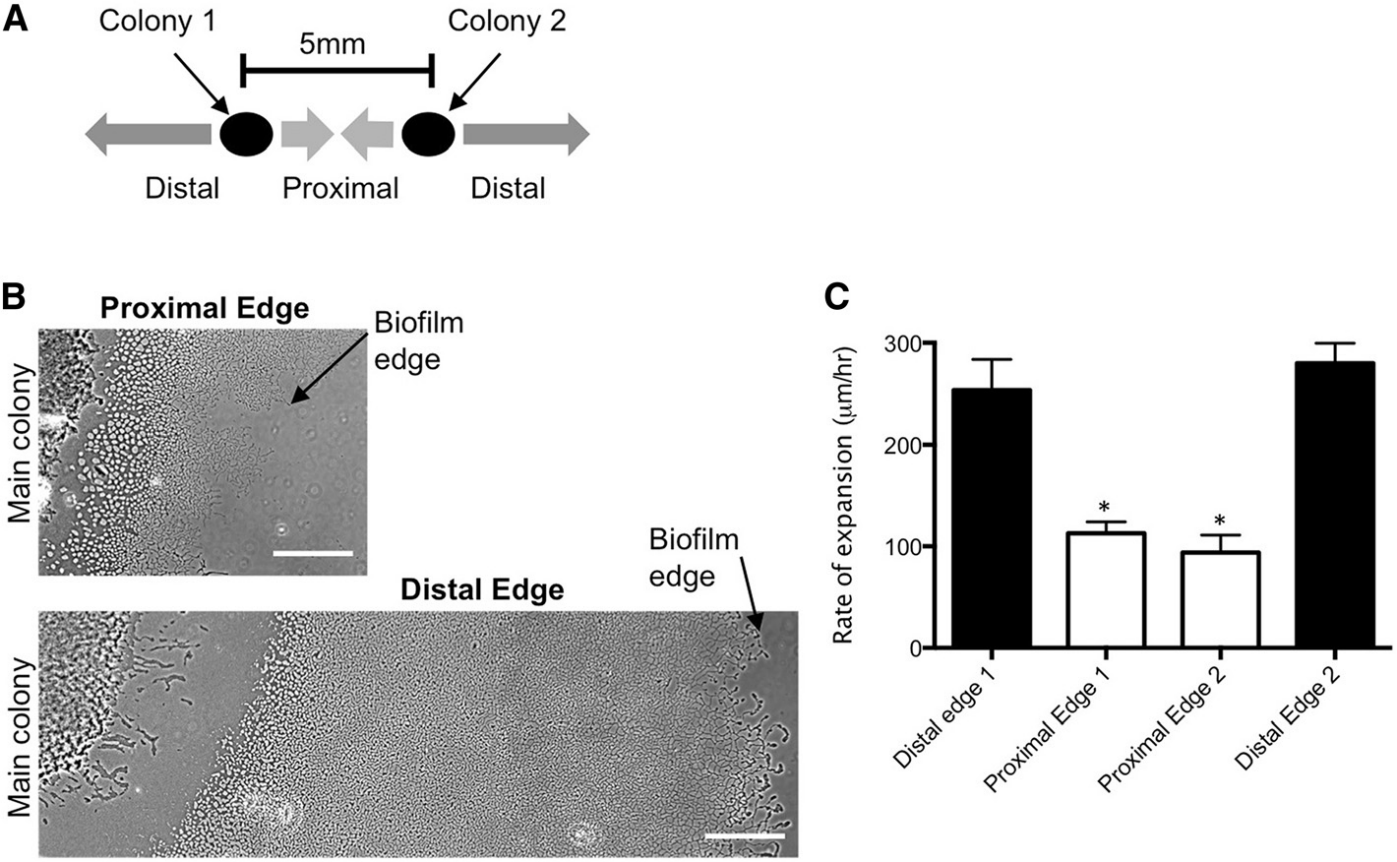
**Self-produced extracellular signals inhibit twitching motility-mediated expansion of** ***P. aeruginosa*** **biofilms.** *P. aeruginosa* PAK inoculated at two adjacent locations 5 mm apart on a solidified media-coated microscope slide results in twitching motility-mediated expansion of two neighbouring biofilms at the interstitial space between the media and coverslip. **(A)** Assay setup: Two *P. aeruginosa* PAK colonies inoculated on a nutrient media-coated microscope slide 5 mm apart expand to form two neighbouring interstitial biofilms. **(B)** Representative phase-contrast microscopy images of *P. aeruginosa* PAK interstitial biofilms formed at the proximal and distal edges of two neighbouring biofilms after incubation at 37°C for 6 h. Scalebar is 200 μm. Images are representative of the twitching motility zones formed in three independent experiments. **(C)** The rate of expansion via twitching motility away from the main colony at the proximal and distal edges of two neighbouring interstitial biofilms after incubation at 37°C for 6 h. Mean ± SD is presented from three independent experiments (two-tailed Student’s *t*-test, *p < 0.05).

We have observed that if *P. aeruginosa* is inoculated twice in neighbouring locations on the same media-coated microscope slide that twitching motility at the proximal edges of each adjacent interstitial biofilm is inhibited, resulting in a significantly decreased rate of expansion of the proximal edges compared to the distal edges (Figure 3). As the proximal edges of these biofilms are about 3–8 mm apart, this phenomenon is unlikely to be due to contact-dependent inhibition. Instead these observations suggest that an endogenously produced extracellular signal(s) such as a small molecule is able to diffuse through the media to repress twitching motility-mediated expansion of the neighbouring biofilm. Given our observations that *P. aeruginosa* colony biofilms contain endogenously produced eATP we hypothesised that these interstitial biofilms may establish a diffused gradient of eATP beyond the colony edge that is sufficient to inhibit twitching motility-mediated expansion of approaching biofilms.

To explore the possibility that gradients of eATP inhibit twitching motility-mediated expansion of approaching biofilms we established an eATP gradient on media coated slides using ATP-saturated filter discs. Filter discs saturated in H_2_O were also used to control for the possible influence of increased water content of the medium surrounding the filter discs. *P. aeruginosa* PAK was then inoculated at a constant distance from the saturated filter discs and a coverslip applied at the edge of the filter disc to establish the interstitial biofilm as per our standard protocol. Time-lapse microscopy was used to examine the effect of the eATP or H_2_O-saturated discs on twitching motility-mediated interstitial biofilm expansion over time (Additional file 2: Movie 2). This movie demonstrates that while cells continue to flow through the trail network to expand the biofilm towards the H_2_O-saturated disc, in the presence of an eATP-saturated disc the cells cease twitching motility.

**Figure 4:**
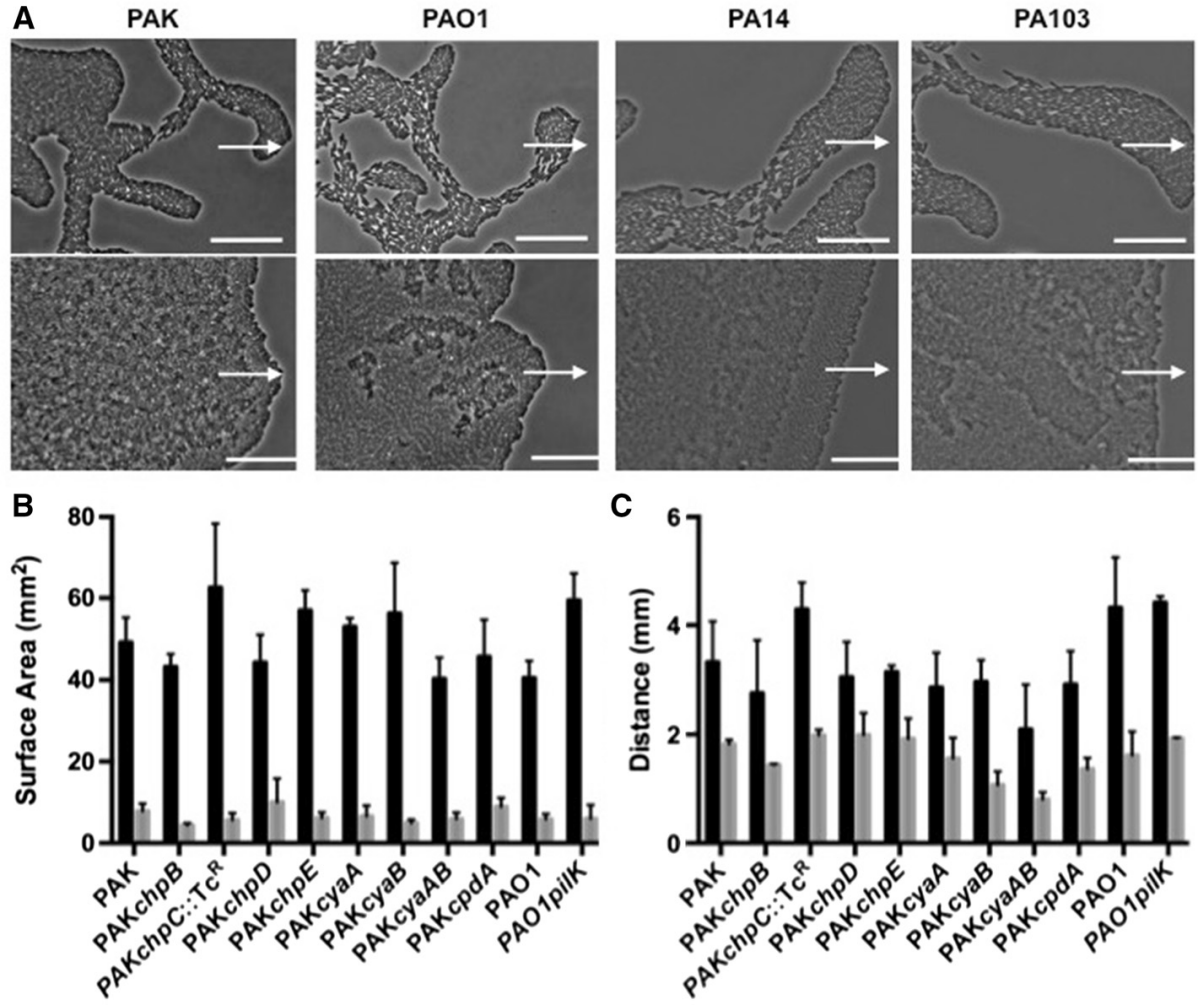
**Examination of twitching motility-mediated biofilm expansion in response to eATP by wildtype** ***P. aeruginosa*** **strains and mutants of** ***cyaA*** **,** ***cyaB*** **,** ***cpdA*** **or the Chp chemosensory system.** The twitching motility response of *P. aeruginosa* strains to eATP at the interstitial space between a solidified nutrient media-coated microscope slide and a coverslip after incubation for 15 h at 37°C. **(A)** Phase-contrast microscopy of interstitial biofilms of wildtype *P. aeruginosa* strains PAK, PAO1, PA14 and PA103 in the presence of a H_2_O-saturated disc (upper images) or ATP-saturated disc (lower images). Scalebar is 50 μm and the arrows indicate the direction of expansion towards the disc. Images are representative of three independent experiments. **(B)** Surface areas of interstitial biofilms formed in the absence of eATP (black bars) or a constant concentration of 7.5 mM eATP (grey bars); **(C)** the distances expanded towards a H_2_O-saturated disc (black bars) or ATP-saturated disc (grey bars). The data are represented as the mean ± SD for three independent experiments.

To determine if the observed inhibition by eATP of twitching motility-mediated biofilm expansion by *P. aeruginosa* strain PAK is likely to be conserved in other *P. aeruginosa* strains, we examined the influence of eATP gradients on interstitial biofilm expansion by the wild-type *P. aeruginosa* strains PAO1, PA14 and PA103. These assays showed all strains were similarly inhibited by gradients of eATP (Figure 4A).

Our observations suggest that *P. aeruginosa* has the capacity to modulate twitching motility-mediated interstitial biofilm expansion in response to a gradient of eATP. *P. aeruginosa* twitching motility is regulated by a putative chemosensory system, the Chp system [22-25] which is homologous to the Che chemosensory system which controls swimming motility of *Escherichia coli* in response to environmental stimuli [26,27]. In *P. aeruginosa* the core signalling components of this system include a putative histidine kinase encoded by *chpA* [22]. ChpA is predicted be coupled to a methyl-accepting chemotaxis protein (MCP) receptor, PilJ, by one of two CheW adaptor protein homologues, PilI and ChpC [22]. This complex is predicted to sense a currently unknown environmental signal which results in PilJ undergoing a conformational change, causing ChpA to be autophosphorylated, with the resulting phosphates being transferred to two CheY-like response regulators, PilG and PilH [23,24]. These phosphorylated CheY-homologs are thought to interact with the tfp motor complex to mediate tfp extension and retraction [22,28,29]. Adaptation to the environmental signal(s) is predicted to be mediated through methylation of PilJ by the competing activities of the methyltransferase PilK and the methylestrase ChpB [22,25]. The Chp system gene cluster also encodes the genes *chpD* and *chpE* which appear to be part of the operon encoding the Chp system but these are not homologous to components of other chemosensory systems and their function have not as yet been determined [22].

It was not possible to examine mutants of the core Chp system components PilG, PilH, PilI, PilJ and ChpA for defects in eATP responses as these mutants are severely defective in twitching motility [22-24]. However, we explored the possibility that the Chp system may be involved in the control of twitching motility in response to eATP by assaying mutants that retain near wildtype levels of twitching motility and tfp assembly [22,25]. Importantly, as PilK and ChpB are predicted to provide the adaptation mechanism that enables responses to a chemical gradient, we hypothesised that if eATP is indeed sensed by this system it is likely that mutants of *pilK* and *chpB* will show defective responses [22,25]. The effects of eATP on the twitching motility of isogenic mutants of the Chp system (*chpB*, *chpC*, *chpD*, *chpE* and *pilK*) were examined in our interstitial twitching motility assay in the presence of 7.5 mM eATP incorporated into the media and in our filter disc gradient assay. These assays revealed that all strains showed similar responses to wild-type to both a constant concentration of eATP and an eATP gradient (Figure 4B,C). This suggests that the Chp chemosensory system is not responsible for control of twitching motility-mediated expansion of interstitial biofilms in response to eATP. However, we cannot rule out the possibility that the core components of the Chp system (PilG, PilH, PilI, PilJ and ChpA) are involved.

Twitching motility is modulated by levels of intracellular cAMP (icAMP) [30-32] which are controlled by the adenylate cyclases CyaA and CyaB that catalyse the conversion of ATP to cAMP, and the phosphodiesterase CpdA which degrades cAMP [33,34]. It is possible that eATP is modulating icAMP via these enzymes. We therefore examined the effect of eATP on the twitching motility of isogenic PAK mutants of the icAMP synthesis/degradation system (*cyaA*, *cyaB*, *cyaAB* and *cpdA*) in our interstitial twitching motility assay in the presence of 7.5 mM eATP incorporated into the media and in our filter disc gradient assay. These assays revealed that all strains showed similar responses to wild-type PAK to both a constant concentration of eATP and an eATP gradient (Figure 4B,C). This suggests that the icAMP synthesis/degradation system is not responsible for control of twitching motility in response to eATP.

### Effects of other extracellular nucleotides on twitching motility

**Figure 5:**
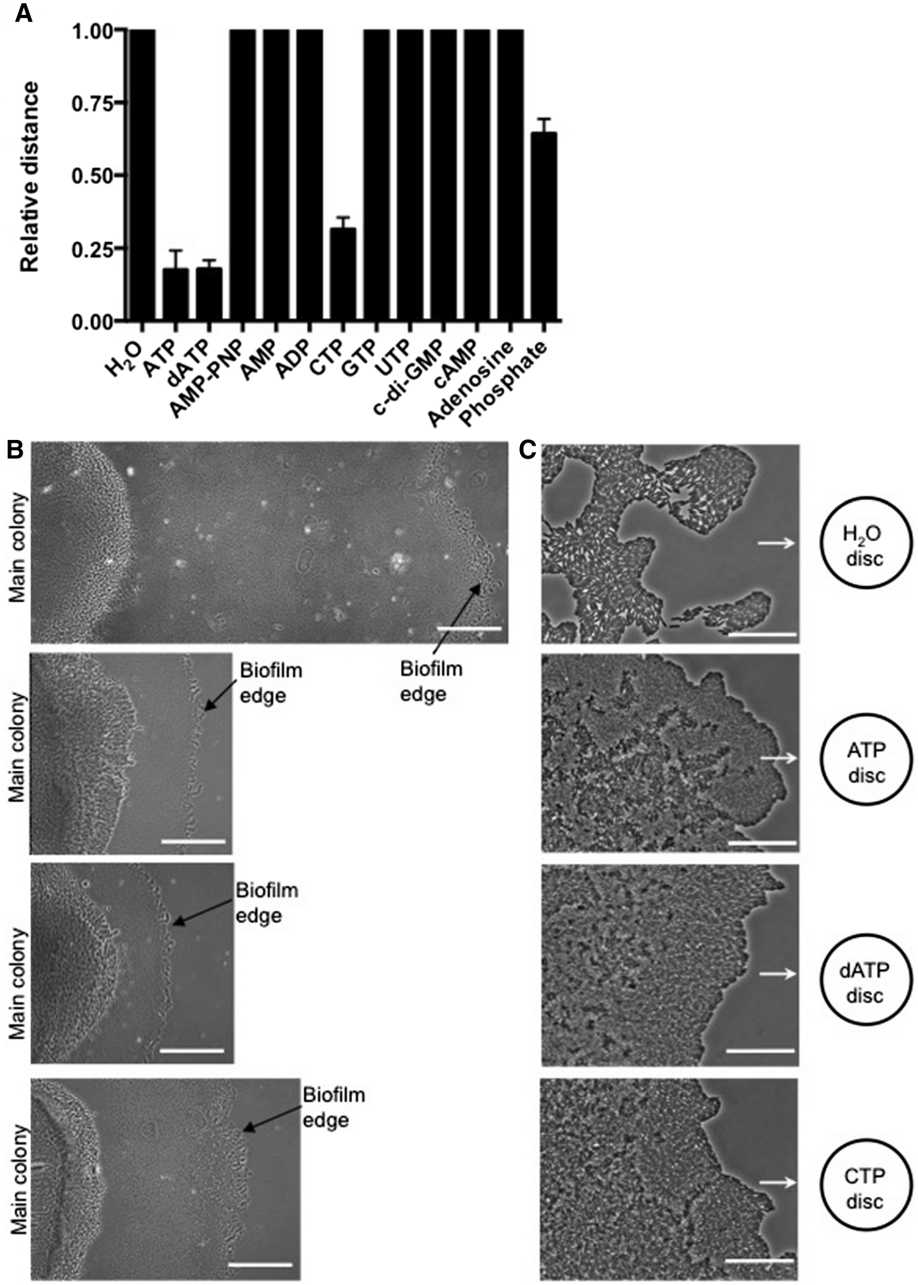
**The effect of a range of extracellular nucleotide signals on twitching motility-mediated expansion of** ***P. aeruginosa*** **biofilms.** Phase-contrast microscopy of interstitial biofilms of *P. aeruginosa* PAK in the presence of a range of nucleotides after incubation for 15 h at 37°C. **(A)** Relative expansion distances of *P. aeruginosa* PAK interstitial biofilms towards a range of saturated discs after incubation for 15 h at 37°C. A value of 1 indicates that *P. aeruginosa* expanded up to the edge of the saturated disc. The data are represented as the mean ± SD from four independent experiments. **(B)** Low magnification images of expansion of the interstitial biofilm away from the main colony towards a H_2_O, ATP, dATP or CTP-saturated disc (scalebar is 500 μm) and **(C)** high magnification images of the biofilm edge closest to the disc (scalebar is 50 μm and the arrows indicate the direction of expansion towards the disc). Images are representative of four independent experiments.

To determine whether the observed inhibition of twitching motility was specific for eATP or whether other nucleotides had equivalent effects, gradients of a range of nucleotides were generated using our filter disc gradient assay. Adenosine monophosphate (AMP), adenosine diphosphate (ADP), guanosine triphosphate (GTP), and uridine triphosphate (UTP), did not have any effect on twitching motility even when the interstitial biofilm had approached very close to nucleotide soaked filter disc (Figure 5). The intracellular second messenger signals bis-(3’–5’)-cyclic dimeric guanosine monophosphate (c-di-GMP) and 3’,5’-cyclic adenosine monophosphate (cAMP), which are involved in controlling a transition between motile and sessile modes of life [35] and in tfp assembly and function [30-32], respectively, also had no effect on twitching motility (Figure 5). Interestingly, deoxyadenosine triphosphate (dATP) and cytidine triphosphate (CTP) had similar inhibitory effects as eATP on twitching motility of the approaching interstitial biofilms (Figure 5).

To determine if the observed inhibition of twitching motility by eATP required hydrolysis of the molecule, we examined the effect of the non-hydrolysable ATP analogue, adenosine-5’-(β,γ-imido) triphosphate (AMP-PNP) in our filter disc gradient assay. We found that the non-hydrolysable ATP analogue did not affect twitching motility-mediated interstitial biofilm expansion indicating that the observed inhibition of eATP on twitching motility requires ATP hydrolysis (Figure 5). We have determined that adenosine, AMP and ADP do not inhibit twitching motility-mediated interstitial biofilm expansion (Figure 5C). Interestingly, phosphate caused a minor inhibition of twitching motility-mediated interstitial biofilm expansion though not to the extent as was observed for either ATP, dATP or CTP. These observations suggest that the twitching motility response of *P. aeruginosa* to eATP is likely to involve an enzymatic process involving hydrolysis of ATP as opposed to direct sensing of the ATP molecule, or its hydrolytic products by a signal transduction system.

## Discussion

In this study we investigated the effect of eATP on twitching motility by *P. aeruginosa*. Our observations have revealed that eATP is inhibitory to twitching motility at concentrations that are not inhibitory to planktonic growth. We found that the presence of eATP results in a reduction in surface assembled tfp levels and that inhibition of twitching motility occurs when eATP is either added at a uniform concentration into the solidified nutrient media or when encountered as a gradient from a source external to the biofilm.

Twitching motility mediated biofilm expansion by *P. aeruginosa* is a complex, multicellular behaviour [9,10] that can lead to the emergence of intricate network patterns of trails that are a characteristic feature of *P. aeruginosa* interstitial biofilms that form under the conditions of our assay. We have recently determined that extracellular DNA and furrow-mediated stigmergy are involved in this self-organisation of the expanding biofilm [10]. However, it remains unclear how individual bacterial cells are able to determine in which direction to travel to enable overall expansion of the biofilm into new territories. We have shown previously that homoserine lactone quorum sensing is not involved in this process [36]. Our observations in this study indicate that eATP is an important extracellular signalling molecule that co-ordinates twitching motility-mediated biofilm expansion of *P. aeruginosa*. Time-lapse microscopy suggests that exogenous eATP at concentrations of 2.5–5 mM may be confounding bacteria within the biofilm so that they were unable to maintain directional movement and that higher eATP concentrations signal the cells to cease movement. We also found that the edge of actively expanding *P. aeruginosa* colony biofilms contain endogenously produced eATP at about 3 mM. Taken together, these observations suggest that a gradient of eATP in the zone of active twitching motility may serve as a signal to direct bacterial traffic toward the virgin territory (low eATP) and away from the older regions of the biofilm (high eATP). High levels of eATP then indicate to the cells that they are no longer near the leading edge and to cease movement.

These observations suggest that a self-produced eATP gradient is sensed by the bacterial cells within the biofilm to direct movement away from regions of high eATP. We examined the possibility that the Chp chemosensory system may be responsible for eATP gradient detection but did not find any evidence that this system is involved. However, as we were unable to examine the core components of this system we cannot completely rule out the possibility. In fact, it is possible that eATP is the central signalling molecule sensed by the Chp chemosensory system to co-ordinate active twitching motility-mediated expansion of the biofilm. Indeed, the observed defects in twitching motility and tfp biogenesis by mutants of these core Chp system components [22-24] are consistent with an inability to detect and respond to eATP.

In this study we determined that ATP hydrolysis appears to be necessary for the inhibition of twitching motility as the non-hydrolysable ATP analogue, AMP-PNP had no effect on *P. aeruginosa* twitching motility-mediated biofilm expansion. However, we found that none of the possible hydrolytic products of ATP (adenosine, AMP, ADP) are inhibitory and that phosphates are only mildly inhibitory to twitching motility. These observations suggest that eATP or its hydrolytic products are not directly sensed by a signal transduction system to control twitching motility-mediated biofilm expansion. Interestingly, a recent study of eATP release by *E. coli* and *Salmonella* sp. in exponential growth phase suggests that this release enhances bacterial survival within stationary phase and that eATP needs to be hydrolysed or degraded at the cell surface [21], which is consistent with our observation that eATP needs to be hydrolysed in order to inhibit *P. aeruginosa* twitching motility-mediated biofilm expansion. As twitching motility is modulated by icAMP levels and icAMP is generated by the enzymatic conversion of ATP to cAMP we assessed whether the icAMP synthesis/degradation system might be responsible for eATP inhibition of twitching motility. However mutants of this pathway showed no defects in the twitching motility response to eATP indicating that this system is not responsible for eATP hydrolysis. Our observation that CTP and dATP also inhibit *P. aeruginosa* twitching motility suggests either that the putative enzyme which hydrolyses eATP is also able to use these nucleotides as substrates or that there is more than one hydrolytic enzyme that is able to hydrolyse eATP. As we did not see a similar inhibition of twitching motility in the presence of UTP and GTP, this suggests that the putative enzyme(s) responsible for the hydrolysis of eATP is unable to readily hydrolyse these nucleotides. It is possible that eATP is used by an unidentified kinase to phosphorylate a key component that controls tfp assembly and twitching motility in *P. aeruginosa*. Interestingly, cAMP and c-di-GMP, two intracellular second messengers which are involved in controlling twitching motility [31,35], did not have any effect on twitching motility when provided exogenously.

*P. aeruginosa* is an opportunistic pathogen that exploits damaged mucosa to cause infection [5,6]. Interestingly, damage to epithelial cells results in a rapid increase in eATP levels, which results in the recruitment of host immune system factors via the P2X and P2Y receptors [16]. Thus it is likely that *P. aeruginosa* is exposed to eATP at the site of epithelial damage. As the cytoplasmic concentration of ATP in mammalian cells is of the order of 5–10 mM, injury causing acute plasma membrane damage is expected to result in the release of eATP concentrations in this range at the injury site [37]. Our observations indicate that eATP above 5 mM is sufficient to inhibit twitching motility by *P. aeruginosa*. This cessation of twitching motility in response to high concentrations of eATP may lead to initiation of infection at the site of epithelial injury. Subsequent cytotoxic damage caused by *P. aeruginosa* may lead to further release of eATP from the damaged epithelial cells. Interestingly, exposure of mammalian cells to mM concentrations of eATP can lead to necrotic or apoptotic cell death [37]. Our observations indicate that the leading edge of *P. aeurginosa* biofilms produce eATP in this cytotoxic range which suggests that eATP produced by *P. aeruginosa* biofilms may be a virulence factor in its own right. Furthermore, chronic *P. aeruginosa* infection of cystic fibrosis patients is associated with significant inflammatory damage and fibrosis [38] and eATP has recently been associated with pulmonary inflammation and fibrosis via the P2X_7_ receptor in a murine model of lung fibrosis [39]. Thus it is conceivable that high levels of eATP produced by *P. aeruginosa* biofilms and through epithelial cell damage contribute to the pathogenesis of chronic *P. aeruginosa* infection.

## Conclusions

This study has shown that eATP produced by *P. aeruginosa* biofilms functions as an extracellular signalling molecule that co-ordinates cell movements during active biofilm expansion. As *P. aeruginosa* is an opportunistic pathogen that infects damaged epithelial tissues it is conceivable that the presence of host-derived eATP at sites of epithelial cell damage is exploited by *P. aeruginosa* to detect potential infection sites. We propose that eATP is an inter-kingdom signalling molecule that contributes to the complex host-pathogen interplay during *P. aeruginosa* infection.

## Methods

### Bacterial strains, plasmids and media

The *P. aeruginosa* strains used in this study are listed in Table 1. *P. aeruginosa* strains were cultured on Luria-Bertani (LB) [40] broth solidified with agar at 1.5% and grown overnight at 37°C. Planktonic cultures were grown in cation-adjusted Mueller Hinton broth (CAMHB) at 37°C, with shaking at 250 rpm. Twitching motility assays were performed with nutrient media (4 g/L tryptone, 2 g/L, yeast extract, 2 g/L NaCl) solidified with 8 g/L GelGro (ICN). ATP (Sigma Aldrich, St Louis, MO) was added at a final concentration of 1.6–15 mM as indicated. *pilK* was inactivated by allelic displacement with a tetracycline resistance cassette (Tc^R^) as described previously [22].

**Table 1:** **List of strains used in this study**

**Strain**	**Relevant characteristics**	**Source or reference**
PAK	Wild-type *P. aeruginosa* strain	D. Bradley, Memorial University of Newfoundlands, St John’s, Canada
PAO1	Wild-type *P. aeruginosa* strain ATCC 15692	American Type Culture Collection
PA14	Wild-type *P. aeruginosa* strain	F. Ausubel, Harvard University, Boston, USA
PA103	Wild-type *P. aeruginosa* strain ATCC 29620	American Type Culture Collection
PAK*pilA*:Tc^R^	*pilA* inactivated by allelic displacement with a tetracycline resistance cassette (Tc^R^)	[19]
PAK*chpB*:Tc^R^	*chpB* inactivated by allelic displacement with a tetracycline resistance cassette (Tc^R^)	[22]
PAK*chpC*:Tc^R^	*chpC* inactivated by allelic displacement with a tetracycline resistance cassette (Tc^R^)	[22]
PAK*chpD*:Tc^R^	*chpD* inactivated by allelic displacement with a tetracycline resistance cassette (Tc^R^)	[22]
PAK*chpE*:Tc^R^	*chpE* inactivated by allelic displacement with a tetracycline resistance cassette (Tc^R^)	[22]
PAO1Δ*pilK* (FA9)	In frame deletion of *pilK* in wildtype strain PAO1	[25]
PAKΔ*cyaA*	In frame deletion of *cyaA*	[33]
PAKΔ*cyaB*	In frame deletion of *cyaB*	[33]
PAKΔ*cyaAcyaB*	In frame deletions of *cyaA* and *cyaB*	[33]
PAKΔ*cpdA*	In frame deletion of *cpdA*	[34]

### Interstitial twitching motility assays

Twitching motility-mediated interstitial biofilm expansion on GelGro-solidified nutrient media was assayed using the solidified nutrient media coated microscope slide assay described previously [18,30]. Interstitial biofilms were examined with phase-contrast microscopy and analysed using FIJI [41].

### Filter disc diffusion assays

Filter discs (Whatman 6 mm, GE Healthcare) were soaked with sterile H_2_O or 100 mM of sodium phosphate or the nucleotides adenosine, adenosine triphosphate (ATP), deoxyadenosine triphosphate (dATP), adenosine monophosphate (AMP), adenosine diphosphate (ADP), cytidine triphosphate (CTP), guanosine triphosphate (GTP), uridine triphosphate (UTP), adenosine-5’-(β,γ-imido)triphosphate (AMP-PNP), 2.9 mM 3’,5’-cyclic diguanylic acid (c-di-GMP) or 30 mM 3’,5’-cyclic adenosine monophosphate (cAMP) and applied to a solidified nutrient media coated microscope slide as follows. 75 μL of the test solution was allowed to fully soak into a filter disc. Each disc was then dried for 2 h and applied to a dried solidified nutrient media coated microscope slide and a gradient allowed to establish for 1 h. The slide was inoculated with the strain of interest 5 mm from the disc, a coverslip applied and incubated at 37°C for the indicated time. In the mutant screening assays each saturated disc was soaked in 30 mM ATP or sterile H_2_O and used as described above.

### Planktonic growth assays

Planktonic growth of *P. aeruginosa* was followed by recording changes in OD_595nm_ for 20 h. Cells were grown in 96-well microtitre plates, and incubated at 37°C, shaking at 250 rpm. CAMHB or CAMHB supplemented with ATP at indicated concentrations was used in growth assays.

### Quantification of eATP in *P. aeruginosa* biofilms

eATP was detected within *P. aeruginosa* surface colony biofilms as follows. 30 mL LB 1.5% agar plates were poured and allowed to set overnight at room temperature. The following morning the agar was flipped into a larger petri dish to expose the smooth underside set against the petri dish base which promotes rapid twitching motility-mediated biofilm expansion [9]. 1.5 mL of an overnight *P. aeruginosa* PAK culture was pelleted by centrifugation (13,000 *g*, 3 min). The pellet was then gently resuspended and inoculated onto the centre of a flipped 1.5% agar. The large petri-dish lid was applied and incubated in a humid environment for 72 h at 37°C. Cells were harvested from the outer, active twitching edge visualised as a “ground-glass edge”. Harvested cells were resuspended in 1 mL PBS and the eATP detected using a BacTitre-Glo™ assay (Promega Corportation). 500 μL of resuspended cells were pelleted by centrifugation (16, 900 *g*, 5 min). 110 μl of supernatant was removed and added into triplicate wells of a white 96-well plate (Greiner Bio-one) and 10 μL used to generate four serial 1:11 dilutions, with each well retaining 100 μL final volume. 100 μL of ATP standards ranging from 0–1 μM were also added to the plate in duplicate. 30 μL of room temperature BacTiter-Glo™ Buffer with precombined BacTiter-Glo™ substrate was added to all wells, mixed and incubated at room temperature for 5 min. Luminescence of all wells was read using an integration time of 250 ms.

### PilA immunoblotting

Preparation of sheared surface tfp and cell-associated pilin samples was performed as described previously [22] with cells being harvested from plates grown at 37°C on LB agar containing 0 mM or 10 mM ATP for 20 h. Samples were displayed on a 10% Bis-Tris Mini Gel (Life Technologies Corporation) and transferred onto an iBlot® mini gel transfer stack containing a PVDF membrane (Life Technologies Corporation). Membranes were probed with a Western Breeze® chemiluminescent western blot immunodetection kit (Life Technologies Corporation) according to manufacturer’s instructions. A 1:5000 dilution of primary anti-PilA antibody was used.

### PilA ELISA

Enzyme-linked immunosorbent assays (ELISAs) to determine the amount of surface tfp of cells grown in the presence and absence of ATP were performed as described previously [22] with cells being harvested from plates grown at 37°C on LB agar containing 0 mM or 10 mM ATP for 20 h.

## Additional files

Additional file 1: Movie 1. Exogenous ATP affects twitching motility of P. aeruginosa. Time-lapse phase-contrast microscopy of *P. aeruginosa* PAK interstitial biofilm expansion on solidified nutrient media-coated microscope slide containing 0 mM, 2.5 mM, 5 mM or 7.5 mM ATP and a coverslip at 37°C. Scalebar is 10 μm. Time (min:s) indicated in top right. Additional file 2: Movie 2. Extracellular ATP gradients affect twitching motility of P. aeruginosa. Time-lapse phase-contrast microscopy of *P. aeruginosa* PAK interstitial biofilm expansion in the presence of an ATP- or H_2_O-saturated disc after 4 h of incubation at 37°C. Scalebar is 10 μm. Time (min:s) indicated in top right.
